# A coupled multimodal planar transmit RF array for ultrahigh field spine MR imaging

**DOI:** 10.1109/TBME.2025.3584217

**Published:** 2026-01

**Authors:** Yunkun Zhao, Komlan Payne, Leslie Ying, Xiaoliang Zhang

**Affiliations:** Department of Biomedical Engineering, State University of New York at Buffalo, Buffalo, NY 14260 USA; Department of Biomedical Engineering, State University of New York at Buffalo, Buffalo, NY 14260 USA; Departments of Biomedical Engineering and Electrical Engineering, State University of New York at Buffalo, Buffalo, NY 14260 USA; Departments of Biomedical Engineering and Electrical Engineering, State University of New York at Buffalo, Buffalo, NY 14260 USA

**Keywords:** Ultra-high field, RF Coil, Surface Coil, B_1_ Efficiency, Specific Absorption Rate, MR imaging

## Abstract

**Objective:**

Ultrahigh-field MRI, such as those operating at 7 Tesla, enhances diagnostic capabilities but also presents unique challenges, including the need for advanced RF coil designs to achieve an optimal signal-to-noise ratio and transmit efficiency, particularly when imaging large samples.

**Methods:**

In this work, we introduce the coupled planar array, a novel technique for high-frequency, large-size RF coil design with enhanced the RF magnetic field (B1) efficiency and transmit performance for ultrahigh-field spine imaging applications. This array comprises multiple resonators that are electromagnetically coupled to function as a single multimodal resonator. The field distribution of its highest frequency mode is suitable for spine imaging applications. Based on the numerical modeling and calculation, a prototype of the coupled planar array was constructed and its performance was evaluated through comprehensive numerical simulations, rigorous RF measurements, empirical tests, and a comparison against a conventional surface coil with the same size and geometry.

**Results:**

The results of this study demonstrate that the proposed coupled planar array exhibits superior performance compared to conventional surface coils in terms of B1 efficiency for both transmit (B1+) and receive (B1−) fields, specific absorption rate (SAR), and the ability to operate at high frequencies.

**Conclusion:**

This study suggests a promising and efficient approach to the design of high-frequency, large-size RF coils for spine MR imaging at ultrahigh magnetic fields.

**Significance:**

The coupled planar array offers a practical solution to overcome scalability and efficiency limitations in ultrahigh-field spine MRI, enabling improved RF performance without requiring complex multi-channel systems.

## I. Introduction

Magnetic Resonance Imaging (MRI) is a pivotal technology for visualizing soft tissues and determining metabolic processes, providing high-resolution images with varied contrasts without employing ionizing radiation [1, 2]. Advancements in MRI technology have led to the development of high and ultrahigh field systems, notably the 7 Tesla MRI, which is now increasingly utilized in clinical settings [3–10]. The transition to a higher static magnetic field strength, from the standard 1.5T or 3T to 7T, significantly enhances the signal-to-noise ratio (SNR) [11–16]. This improvement not only boosts image resolution and spectral dispersion, but also reduces scan times, utilizing the parallel imaging [17–20] and compressed sensing based fast imaging techniques[21–23]. Additionally, ultrahigh field MRI enhances vasculature conspicuity, improves angiography, and augments spectroscopy acquisitions[24–27].

Despite the substantial benefits of ultrahigh field MRI, the required high resonance frequency of the ultrahigh field, e.g. ~300MHz at 7T, poses significant challenges in designing large transmit RF coils for MR signal excitation, which can impede its optimal performance and functionality [28–34]. The key requirements in designing large transmit RF coils in ultrahigh field MRI are (1) high efficiency of transmit RF magnetic field (B1+), (2) ability to achieve high resonant frequency, and (3) low specific absorption rate (SAR). Large size RF coils, e.g. large L/C loops, inherently face difficulties in achieving high transmit efficiency due to their physical dimensions and the circuit layout. Due to their intrinsically high inductance, the large transmit coils suffer from achieving the required high resonance frequency. To overcome the inductance challenges and operate at the required high frequency, the capacitance of large transmit coils must be significantly reduced. However, reducing capacitance can generate high electric fields, potentially increasing the local SAR and tissue heating, ultimately posing safety hazards to patients or subjects being imaged. Due to these technical challenges, currently no large size transmit RF coils are available in clinical ultrahigh field MR systems.

In recent years, methods using multichannel transmit or transceive arrays to excite large samples, e.g. the spine, at the ultrahigh field of 7T have been explored [35–38]. High-channel-count phased array coils, such as 8-channel transmit arrays, have demonstrated enlarged excitation coverage and multichannel transmit capability compared to earlier single-channel designs for cervical spinal cord imaging at 7T [36, 37]. However, imaging with multichannel transmit arrays requires high-power multichannel transmitters [39], which are not cost-effective and are often unavailable in standard MR scanners. Developing a technique for designing large, single-channel, and highly efficient transmit coils would be highly beneficial for ultrahigh field MR imaging applications.

To address the challenges encountered in the design of large transmit coils at ultrahigh fields, in this work, we propose and investigate a novel solution: the coupled planar RF array for high-frequency large-size transmit coils [40]. This design comprises multiple L/C loop resonators, which are arranged in a planar array with no overlap. All the resonators are electromagnetically coupled, thereby forming a single multimodal resonator. Among those multiple resonant modes, the one resonating at the highest frequency exhibits a distinctive magnetic field distribution, analogous to that of conventional loop surface coils and is therefore suitable for MR imaging. The experimental results demonstrate that the proposed coupled planar array outperforms the conventional surface coils in spine imaging at the ultrahigh field of 7T in terms of high-frequency operation, B1 efficiency and penetration, and SAR. This coupled planar array technique provides a simple and robust solution to designing high-frequency large-size transmit coils for ultrahigh field spine MR imaging with improved transmit efficiency and lower SAR, which ultimately enhances the functionality and patient safety of ultrahigh field MR.

## II. Description of Methodology

### A. EM Simulation

**Fig. 1.**
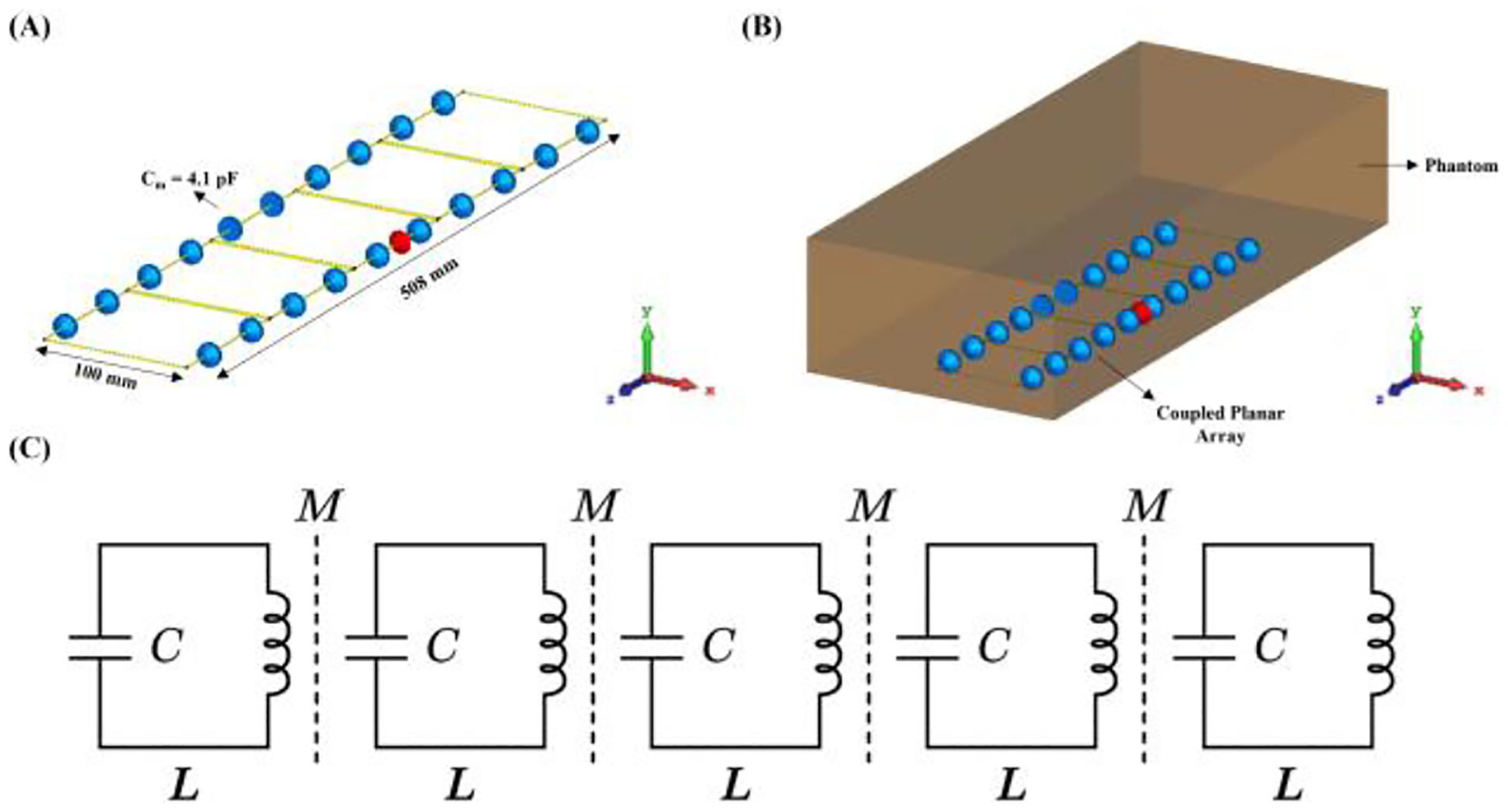
(A) Simulation model of the proposed coupled planar array, showing the dimensions and detailed structure. (B) Proposed coupled planar array loaded with a cuboid phantom. (C) Equivalent circuit model of coupled planar array. Each resonator comprises an inductor L (self-inductance) in series with a capacitor, C (tuning capacitance); nearest-neighbor coupling is represented by mutual inductance M (magnetic coupling) between each pair of adjacent inductors (dashed lines).

Figure 1 presents the simulation model of the coupled planar array. This array consists of five resonators or coil loops, each constructed from 16 AWG copper wire and sequentially arranged into a coil array. Four of these coils are identical square units, each measuring 10 cm on each side and equipped with four 4.1pF capacitance tuning capacitors to tune the resonators to 263 MHz. When electromagnetically coupled, the array operates at a higher resonant frequency of 300 MHz, which is achieved through the combined effects of the coupled design. The central coil includes a driving port and an impedance matching circuit, crucial for the operation of the coupled planar array. The coils are spaced only 1 mm apart to maximize mutual inductive coupling, with the total length of the array extending to 50 cm and a width of 10 cm. Designed with multiple coils, our array supports three resonant modes. We utilize the highest resonant mode for imaging applications, chosen for its ability to produce a uniform B1 field direction and reach high frequencies effectively. In this study, we evaluated the performance of the proposed coupled planar array against two reference structures to establish comparative benchmarks. First, a large single-loop conventional surface coil was constructed using 16 AWG copper wire and shares the same overall footprint as the coupled array, measuring 50 cm in length and 10 cm in width. It was tuned to operate at 300 MHz using twelve evenly distributed 3.2 pF capacitors. Second, a decoupled overlapped loop array was designed and evaluated: it consists of five individual 10 cm × 10 cm loops arranged to match the same overall dimensions, each tuned to 300 MHz and modeled with geometric overlap for decoupling. Each loop was impedance-matched to 50 ohms. The decoupled overlapped loop array is driven from a single power source via an ideal (lossless) power splitter. Both reference designs serve as benchmarks for assessing B1 field efficiency under identical operational conditions alongside the coupled planar array. In simulation, all designs are placed 1 cm below an oil phantom with a dimension of 70×30×15 cm2. An alternative simulation comparison has also been applied using CST body model Gustav. The bio model has also been placed 1 cm above the coil designs. Performance assessments of the study involved analyzing scattering parameters, SAR, and B1 efficiency, using field distribution plots. All electromagnetic field plots were normalized to 1 W of total accepted power. Numerical results of the proposed designs were obtained using the electromagnetic simulation software CST Studio Suite (Dassault Systèmes, Paris, France).

### B. Bench Test Model Assembly

**Fig. 2.**
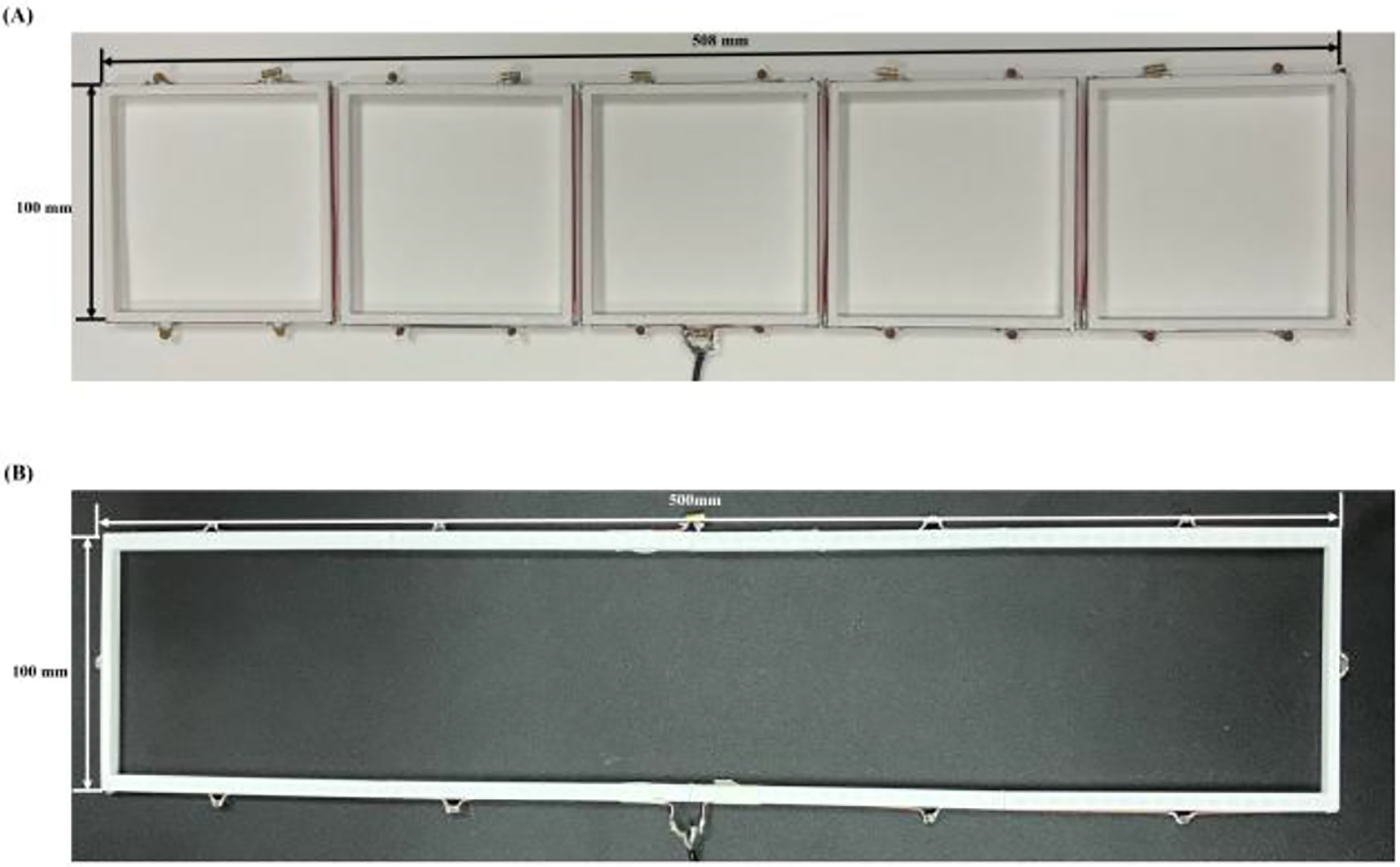
Bench test model of (A) coupled planar array and (B) large, single-loop conventional surface coil.

Figure 2 presents photographs alongside dimensional details of the bench test models for our coupled planar array and the comparative designs. These test models retain the same dimensions as specified in the simulation models, ensuring consistency across experimental and simulated setups. The coupled planar array was constructed with 16 AWG copper wire and built upon a 3D printed polylactide structure fabricated using a Flashforge Guider 2s 3D printer (Flashforge, Zhejiang, China). The array was meticulously tuned to a resonant frequency of 298 MHz, aimed at matching the operational frequency of 7T ultrahigh field MRI systems. The array incorporates twenty identical 4pF capacitors, ensuring uniformity and reliability in performance.

A large, single-loop conventional surface coil was constructed for comparison purposes. The coil was built using 16 AWG copper wire and tuned with eleven 1.8 pF capacitors. To ensure structural stability and maintain consistency with the coupled planar array, the coil was also mounted on a 3D-printed polylactide structure. This setup facilitated a direct and reliable performance comparison with the proposed coupled planar array.

**Fig. 3.**
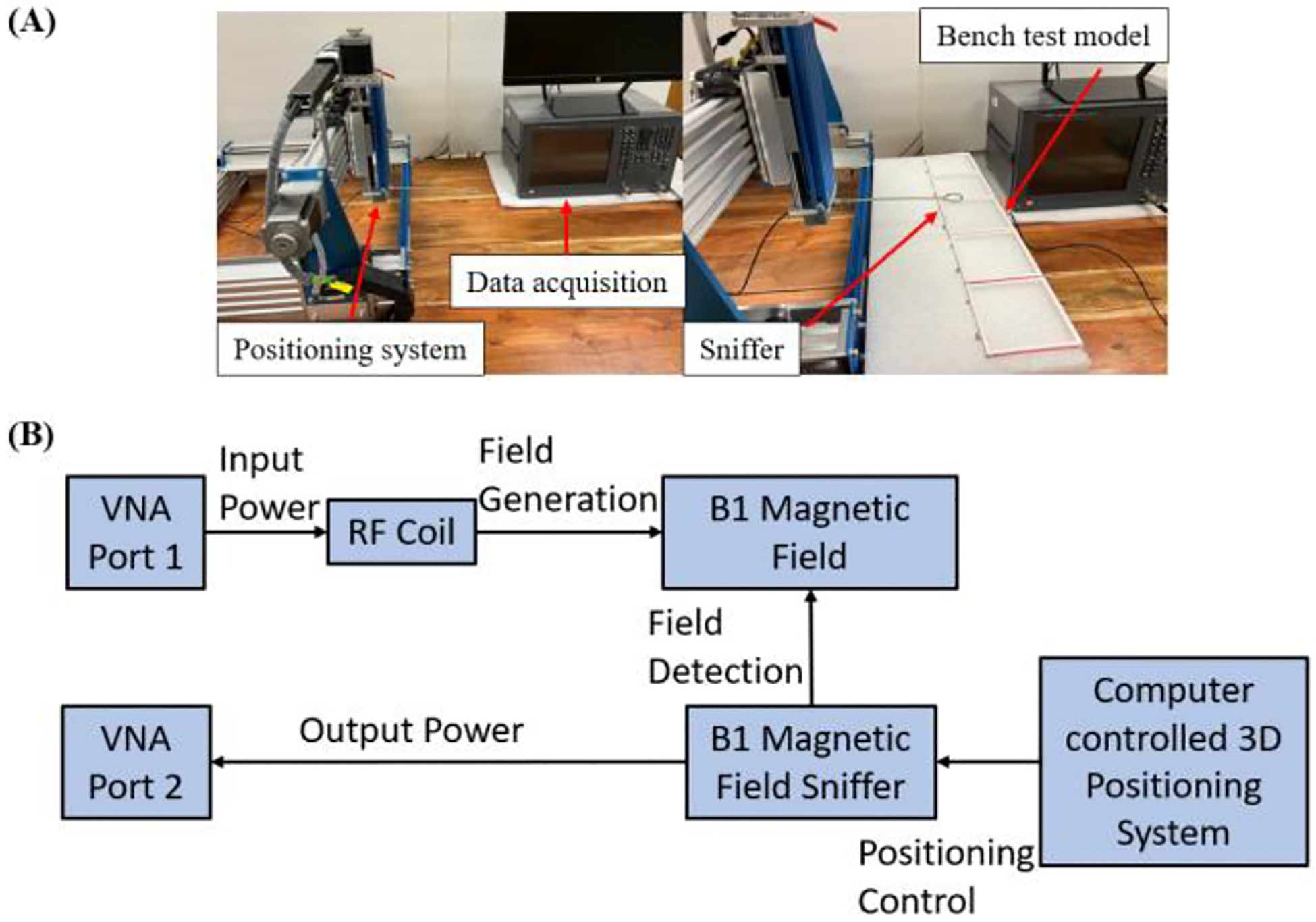
(A) Photograph of measurement setup including the sniffer-positioning system, network analyzer and the data process computer. (B) Schematic of the measurement workflow to obtain B1 field efficiency map.

As shown in Figure 3, the experimental configuration incorporates an H-field sniffer, which is mounted on a high-precision router system, the Genmitsu CNC PROVerXL 4030. This setup facilitates the precise placement of the field probe to capture the B1 field emitted by the RF coil in a three-dimensional space. This probe is connected to a Keysight E5061B vector network analyzer (Santa Clara, CA, U.S), which collects the raw data including output, accepted power, and scattering parameters. The data captured by the vector network analyzer is then transmitted to a computer and analyzed using MATLAB to generate the B1 field efficiency map. The efficiency mapping is conducted on a 30 × 7 cm slice in the Y-Z plane, positioned 1 cm above the RF coils, with measurements taken every 2.5 mm. All experimental results are normalized to 1 watt of accepted power to standardize the output across tests.

### C. Theoretical Analysis of a 5-Coil Coupled System

In our design, we consider a linear array of five identical LC loops (RF coils) that are coupled only to their nearest neighbors. Because each loop is identical, the uncoupled (natural) angular frequency of each LC resonator is

$$\omega_{0}=\frac{1}{\sqrt{LC}}$$

When the coils are brought into close proximity, mutual inductive coupling introduces interactions among them. For a pair of coils with self-inductances L and mutual inductance M, the coupling coefficient is defined as

$$k=\frac{M}{L}$$

Assuming that only nearest–neighbor coupling is significant, the dynamics of the N-coil system can be modeled by the following set of coupled equations:

$$\left\{\begin{array}{c} \left(\omega_{0}^{2}-\omega^{2}\right)I_{1}+k\omega_{0}^{2}I_{2}=0\\ k\omega_{0}^{2}I_{1}+\left(\omega_{0}^{2}-\omega^{2}\right)I_{2}+k\omega_{0}^{2}I_{3}=0\\ k\omega_{0}^{2}I_{2}+\left(\omega_{0}^{2}-\omega^{2}\right)I_{3}+k\omega_{0}^{2}I_{4}=0\\ \vdots \\ k\omega_{0}^{2}I_{N-1}+\left(\omega_{0}^{2}-\omega^{2}\right)I_{N}=0 \end{array}\right.$$

This system can be expressed in matrix form as

$$\left(\begin{array}{ccccccc} \omega_{0}^{2}-\omega^{2} & k\omega_{0}^{2} & 0 & \cdots & \cdots & \cdots & 0\\ k\omega_{0}^{2} & \omega_{0}^{2}-\omega^{2} & k\omega_{0}^{2} & 0 & \cdots & \cdots & 0\\ 0 & k\omega_{0}^{2} & \omega_{0}^{2}-\omega^{2} & k\omega_{0}^{2} & 0 & \cdots & 0\\ \vdots & \vdots & \vdots & \vdots & \vdots & \vdots & \vdots \\ 0 & \cdots & \cdots & \cdots & 0 & k\omega_{0}^{2} & \omega_{0}^{2}-\omega^{2} \end{array}\right)\left(\begin{array}{c} I_{1}\\ I_{2}\\ I_{3}\\ \vdots \\ I_{N} \end{array}\right)=\left(\begin{array}{c} 0\\ 0\\ 0\\ \vdots \\ 0 \end{array}\right)$$

Nontrivial solutions for the currents I exist only if the determinant of the coefficient matrix is zero. For a linear chain of N coupled oscillators with only nearest–neighbor interactions, it is well known that the eigenvalue problem yields

$$\omega_{n}^{2}=\omega_{0}^{2}{\left[1+2k\text{cos}\;\left(\frac{n\pi }{N+1}\right)\right]}^{-1},n=1, 2, 3,\ldots ,N$$

For our five-coil system (N=5), the eigenfrequencies become

$$\omega_{n}^{2}=\omega_{0}^{2}{\left[1+2\text{kcos}\;\left(\frac{n\pi }{6}\right)\right]}^{-1},n=1, 2, 3, 4, 5$$

Because each value of n yields a distinct cosine value, the coupling splits the original frequency $\omega_{0}$ into five distinct eigenfrequencies.

### D. Current Distribution and Mode Field Directions

The spatial distribution of the coil currents (which determine the field directions) is given by the eigenvectors of the coupling matrix. For a chain of N=5 coils with open boundaries, the relative current (and thus field) pattern in coil j for mode i is given by the eigenvector component

$$I_{j}^{\left(i\right)}\propto \text{sin}\;\left(\frac{jn\pi }{N+1}\right),j=1, 2, 3,\ldots ,N,i=1, 2, 3,\ldots ,N$$

The sign of each component of the eigenvector indicates the relative phase (and hence the current direction) in each coil. Choosing a reference direction (for example, defining a positive current as clockwise), a negative value indicates the current flows in the opposite (counterclockwise) direction. Below we summarize the eigenvector frequencies and patterns for each mode:

## III. Result

### A. Simulated Resonant Frequency and Field Distribution

**Fig. 4.**
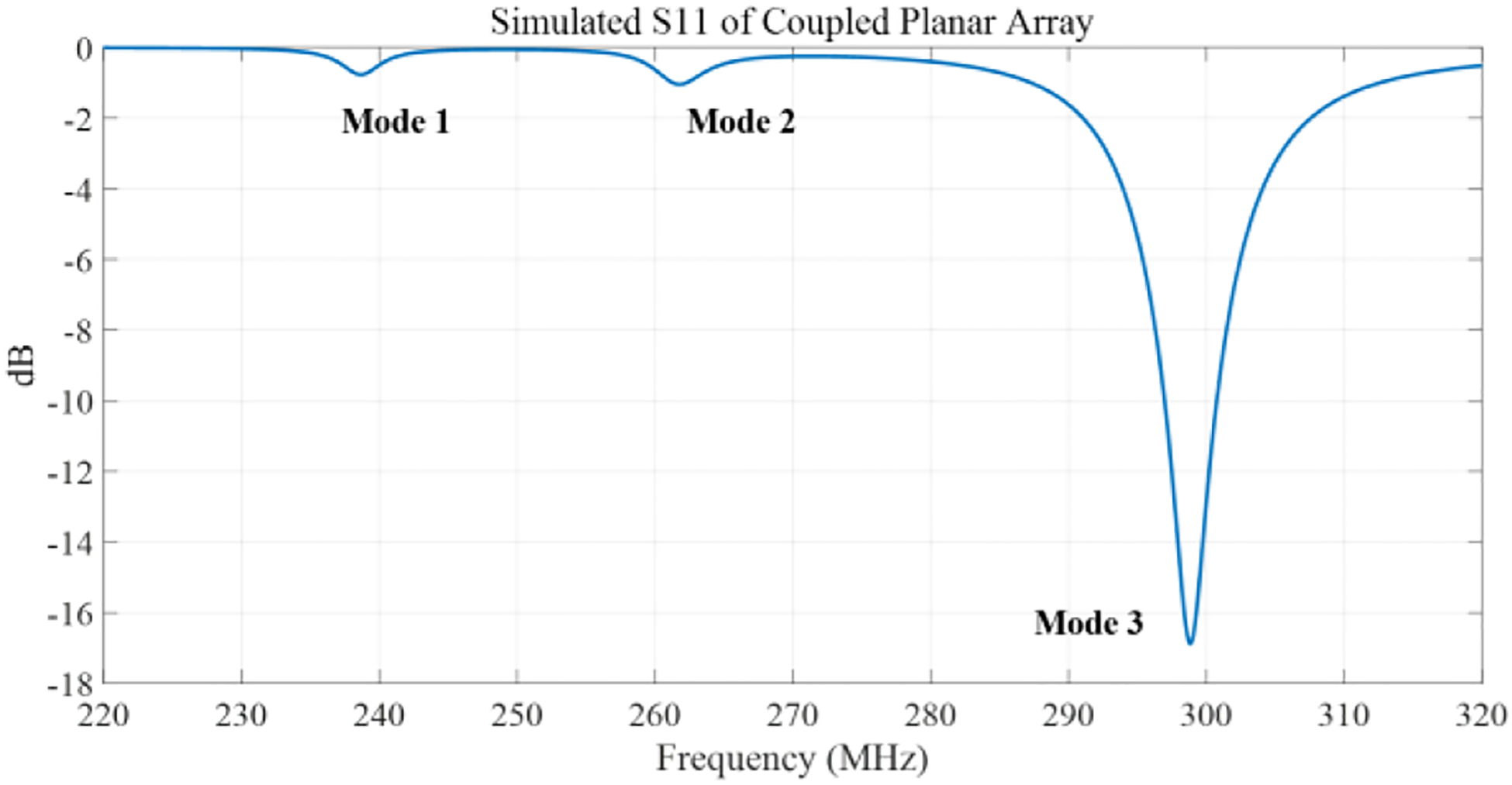
Simulated reflection coefficient S11 vs. frequency of the coupled planar array. Three resonant modes with frequencies of 238 MHz, 261 MHz, and 298 MHz were observed.

**Fig. 5.**
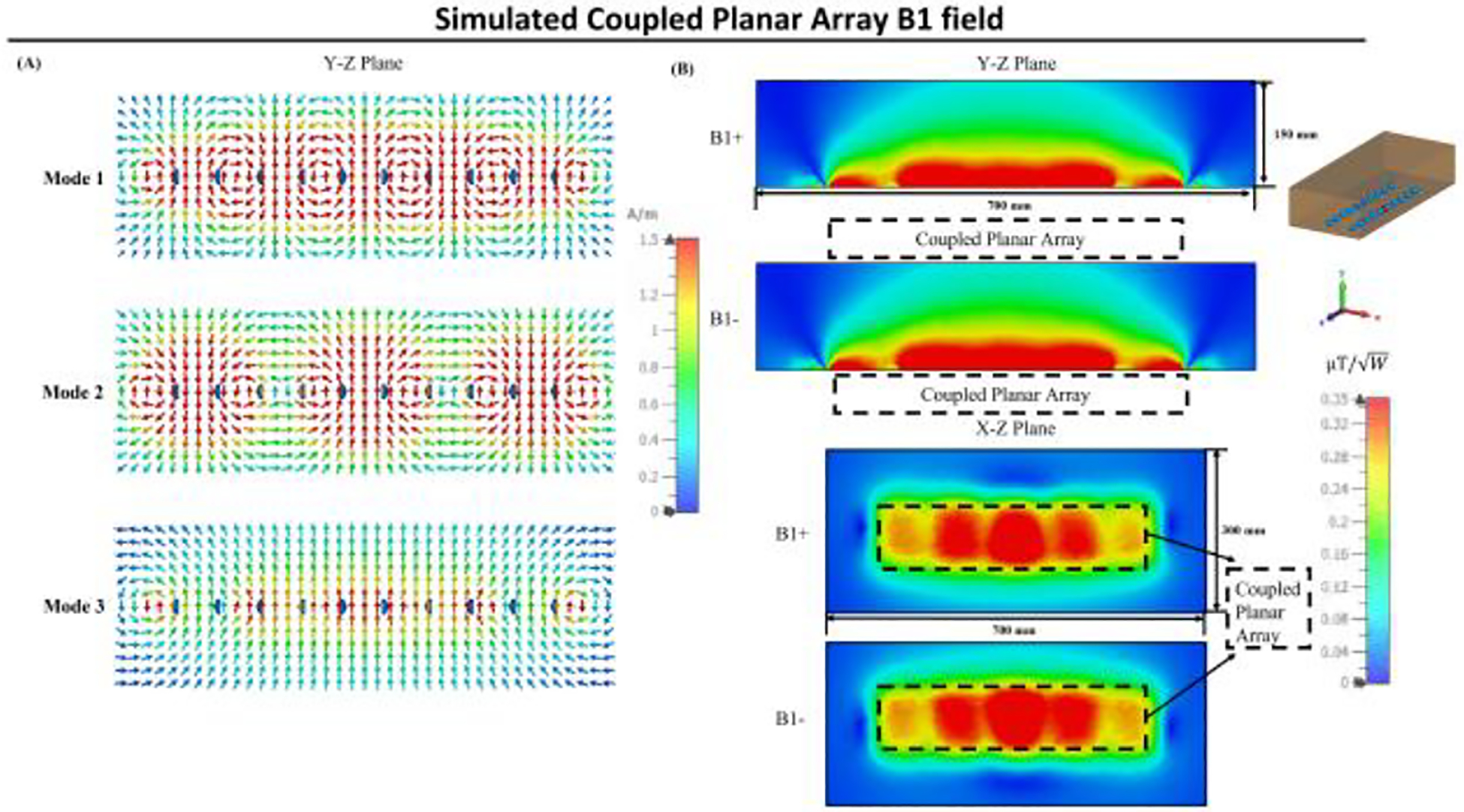
(A) Magnetic field direction and distribution for all three resonant modes of the coupled planar array. (B) Simulated Y-Z and X-Z plane B field efficiency maps inside phantom generated by coupled planar array.

Figure 4 illustrates the simulated scattering parameters vs frequency for the coupled planar array. The figure displays three distinct split resonant peaks at 238 MHz, 261 MHz, and 298 MHz, with the highest peak at 298 MHz designated for imaging applications. Notably, each coil within the coupled planar array resonates naturally at a lower frequency of 263 MHz, highlighting the success of our tuning strategy that utilizes a higher capacitance value to achieve increased resonant frequencies for coils of equivalent size. Figure 5A illustrates the magnetic field direction and distribution for all three resonant modes of the coupled planar array. The highest frequency mode demonstrates a field pattern analogous to that of a conventional surface coil. In Figure 5B, the B1 field efficiency maps are shown across the Y-Z and X-Z planes inside the phantom. The Y-Z plane field maps are taken through the center of both the coil and the phantom, while the X-Z plane corresponds to a horizontal slice located 3.5 cm below the coil surface, within the central region of the phantom. These maps depict the B1 field efficiency of the coupled planar array, demonstrating its capability to generate a strong and well-distributed B1 field.

**Fig. 6.**
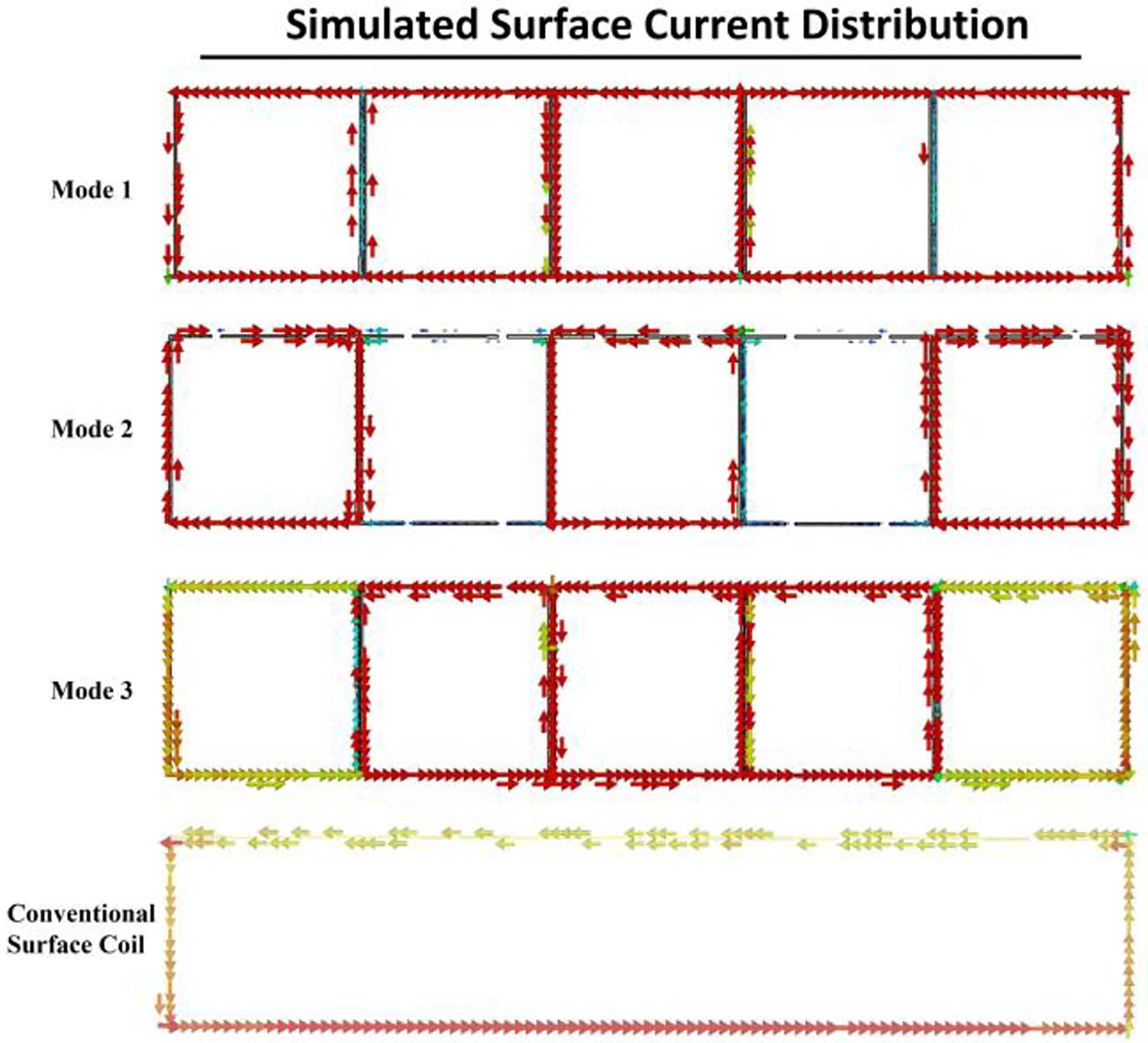
Simulated surface current distribution plot of the multimodal surface coil and conventional surface coil. Coil 1 is positioned at the left rear side, with each subsequent coil (Coil 2 through Coil 5) positioned progressively to the right, culminating in Coil 5 at the rear right. Coil 3 is designated as the driving coil.

Figure 6 depicts the simulated distribution of surface currents across each individual coil. At mode 3, the currents flow in a uniform counterclockwise direction throughout the coils, reflecting a consistent and effective driving field. An increment in the magnitude of surface current is particularly noticeable in the central driving coil (Coil 3), decreasing progressively toward the peripheral coils. This pattern of current intensity can be attributed to the electromagnetic coupling within the coils, with the central coil directly connected to the power source, inducing more robust currents. As distance from the center increases, the influence of the driving coil diminishes, a result consistent with the increasing impedance and potential energy losses encountered as the current moves through the planar coupled resonant structure.

### B. Measured Scattering Parameters and Field Distribution

**Fig. 7.**
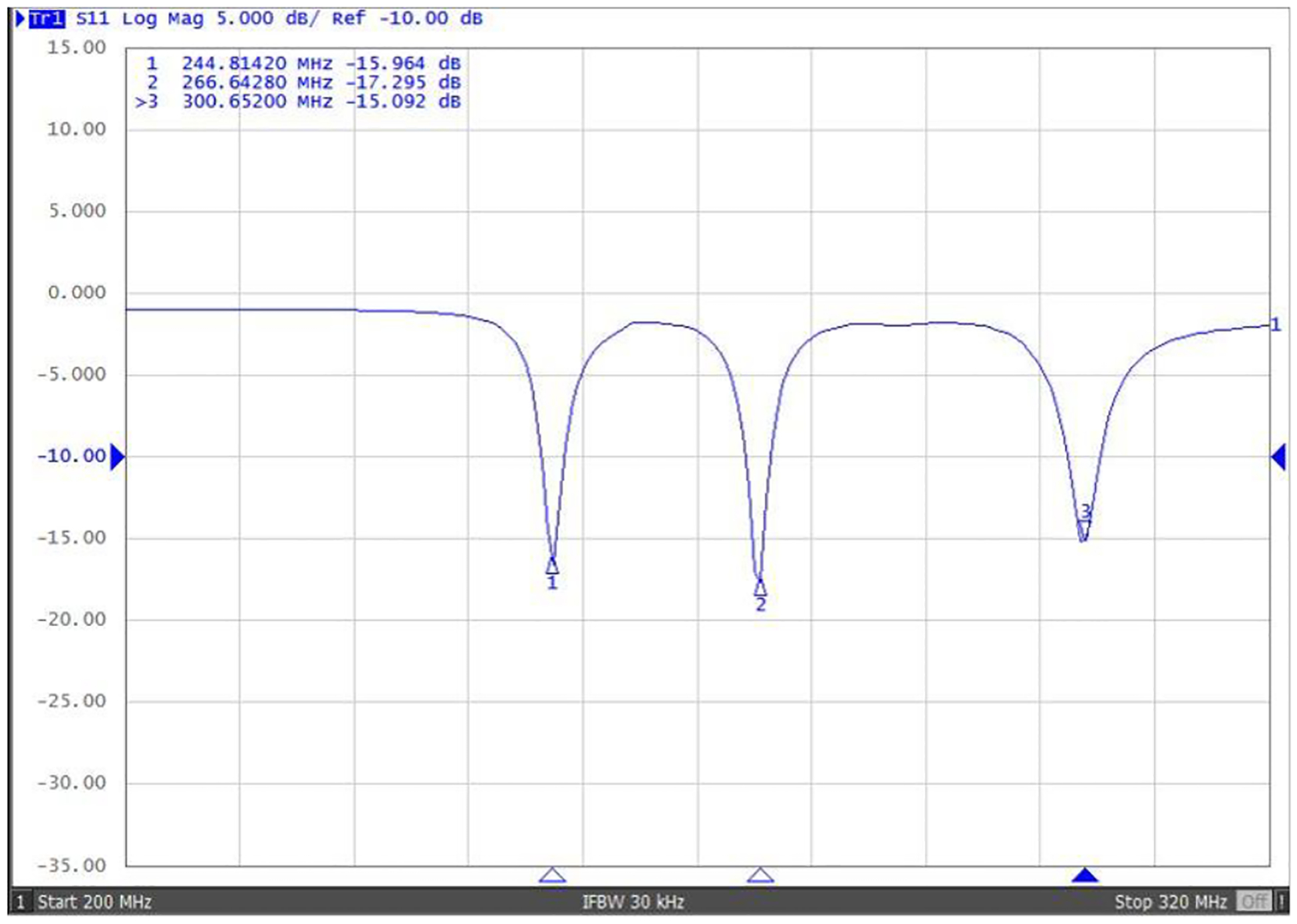
S11 reflection measurement vs. frequency of the bench test model of coupled planar array.

**Fig. 8:**
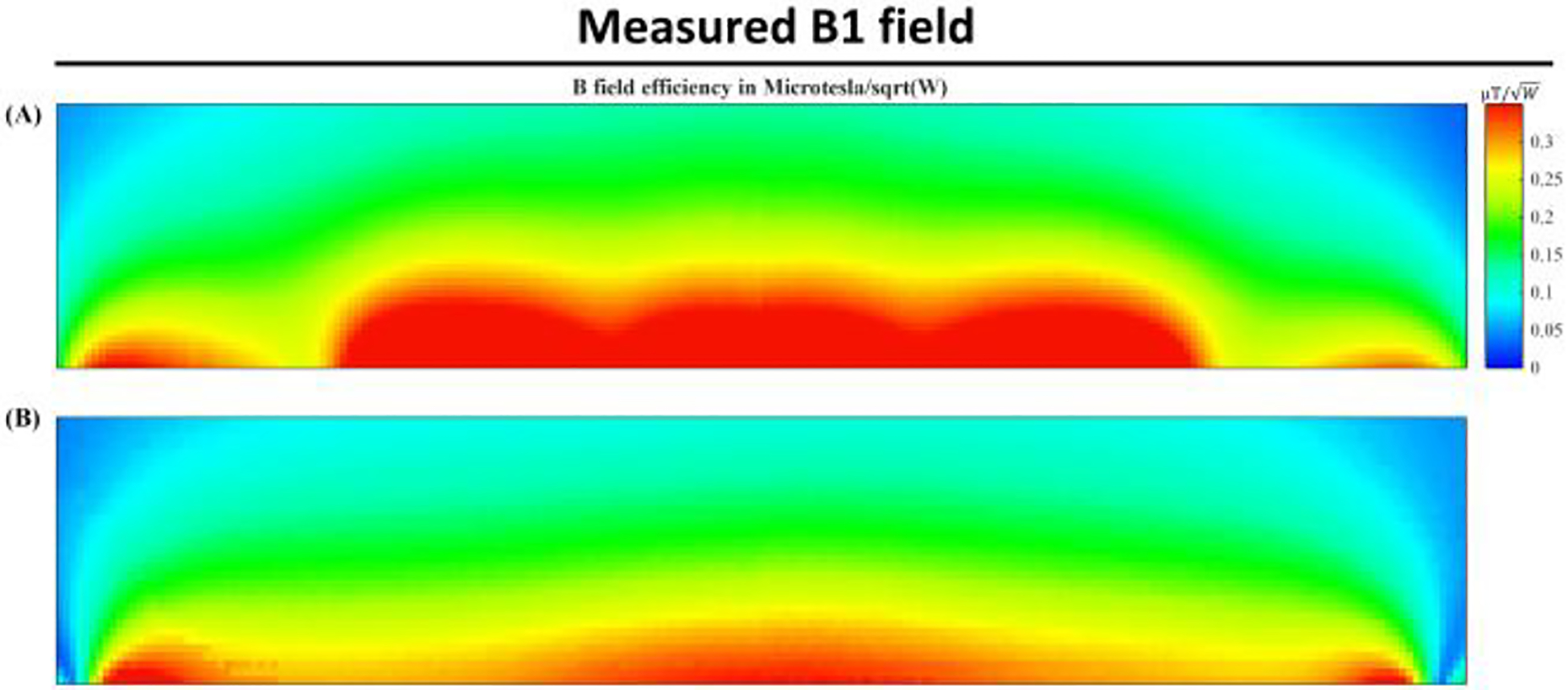
Experimental B1 field mapping of the (A) coupled planar array and (B) conventional surface coil.

Figure 7 displays the S-parameter versus frequency plots for the coupled planar array, demonstrating a close alignment with the predicted simulation outcomes. This figure reveals the formation of three resonant modes at frequencies of 244.8 MHz, 266.6 MHz, and 300.6 MHz. Figure 8 illustrates the B1 field efficiency distribution map on the Y-Z planes, obtained using a 3-D magnetic field mapping system. The coupled planar array exhibits robust B field efficiency and maintains a consistent field distribution pattern across the Y-Z planes, corroborating the simulation results. This congruence underlines the precision and reliability of the simulations, confirming the effectiveness of the coupled planar array in achieving expected performance metrics.

### C. Field Distribution and Efficiency Evaluation

**Fig. 9:**
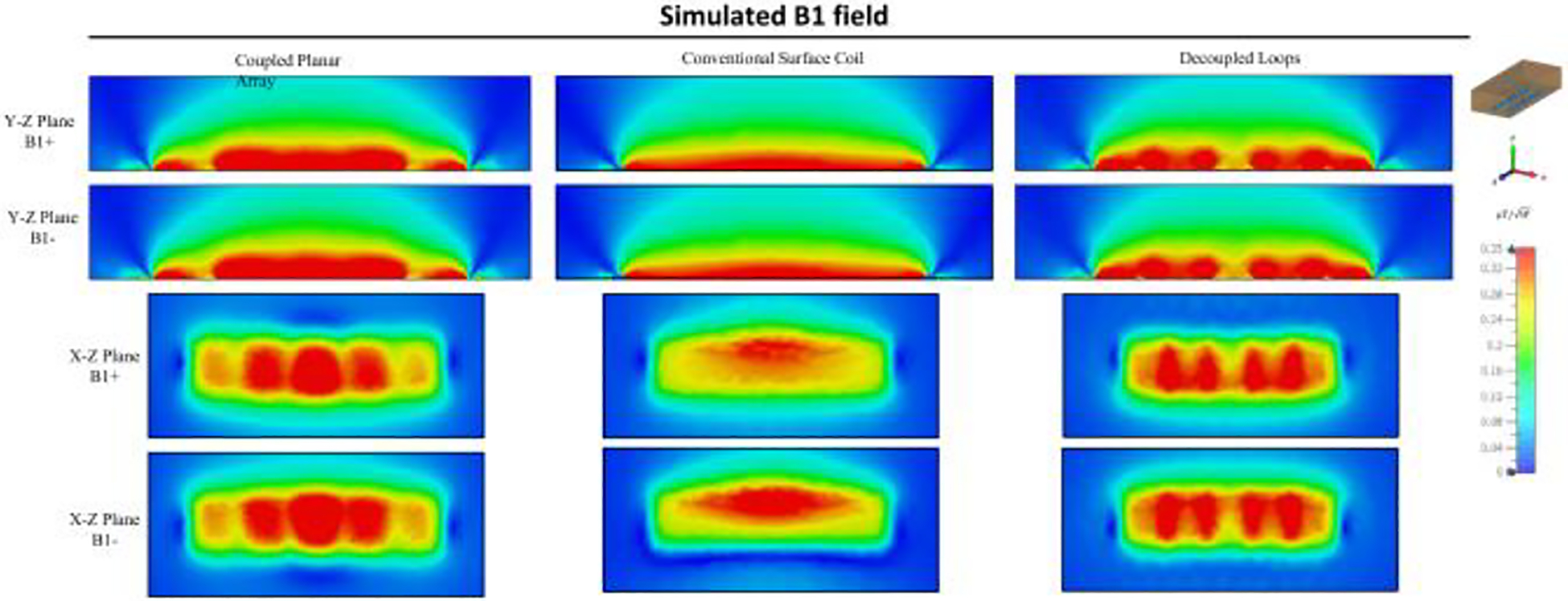
Simulated Y-Z and X-Z plane B1 field efficiency maps inside phantom generated by coupled planar array, conventional surface coil, and decoupled loops. X-Z planes are at the center of the coil and X-Z plane is 3.5 cm above the coil.

**Fig. 10:**
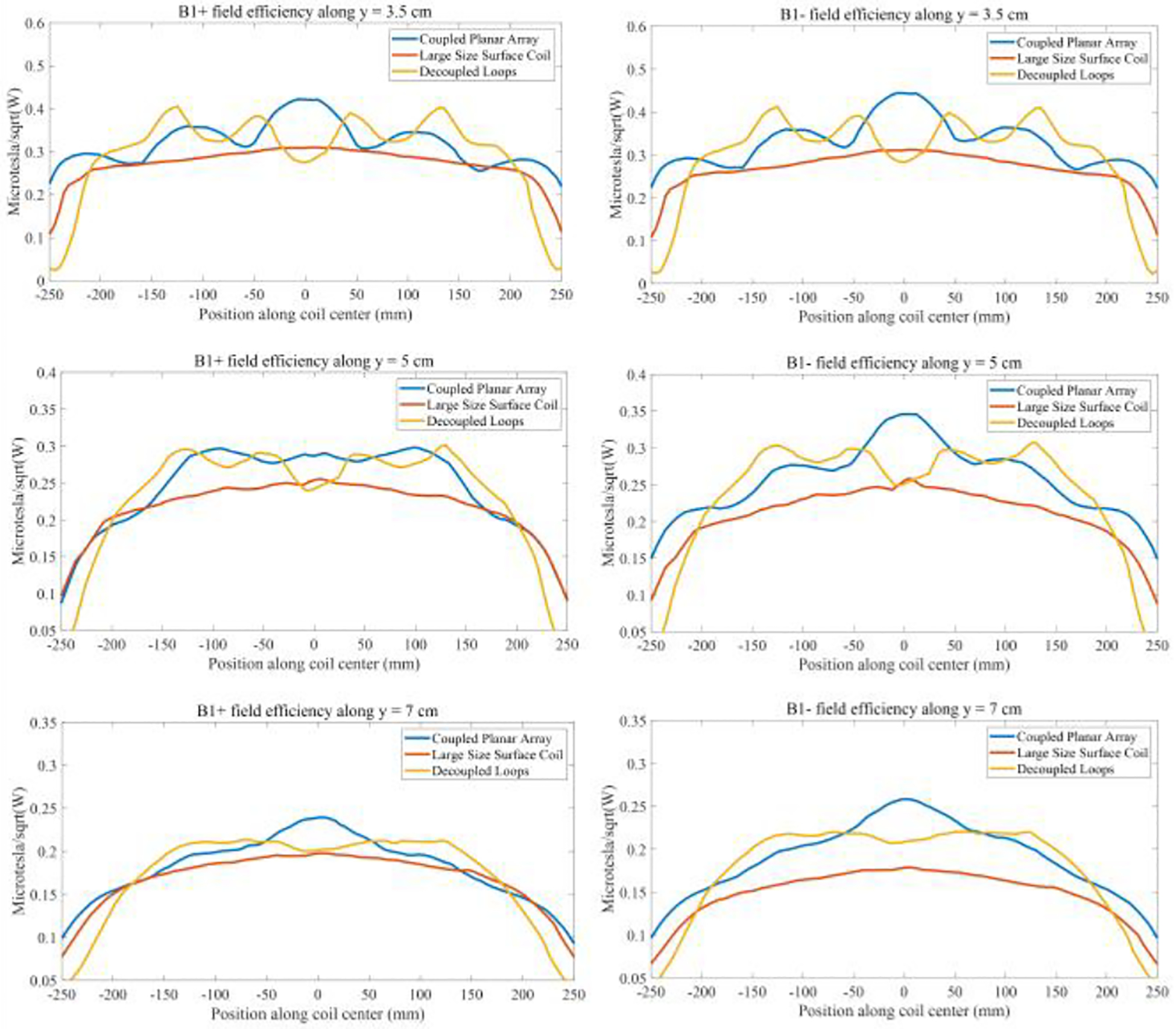
1-D plot of B1 field efficiency along the horizontal line y = 3.5 cm, y = 5 cm, and y = 7 cm.

Figure 9 offers a detailed comparative analysis of the simulated B1 field efficiency between the coupled planar array, a conventional large-size surface coil, and decoupled loops. The findings clearly illustrate that the coupled planar array achieves a significantly higher B1 field efficiency compared to its counterparts. In Figure 10, a one-dimensional plot of the B1 field efficiency is presented along a horizontal line positioned 3.5 cm, 5cm, and 7 cm above the coils. At y = 3.5 cm, y = 5 cm, and y = 7 cm, the B1 field efficiency of the coupled planar array is 20.5%, 17.1%, and 17.6% higher compared to the conventional surface coil. Moreover, its performance exceeds that of the decoupled loops by 7.4%, 3.6%, and 4.7% at these same depths, without accounting for insertion losses of the required power splitter and/or Butler matrices. It is evident from the plot that the average B1 field efficiency achieved by the coupled planar array markedly exceeds that of the conventional surface coil and decoupled loops. Such results underscore the advanced capabilities of the coupled planar array in delivering stronger B1 field across a given area, which is essential for achieving consistent and high-quality imaging results.

**Fig. 11:**
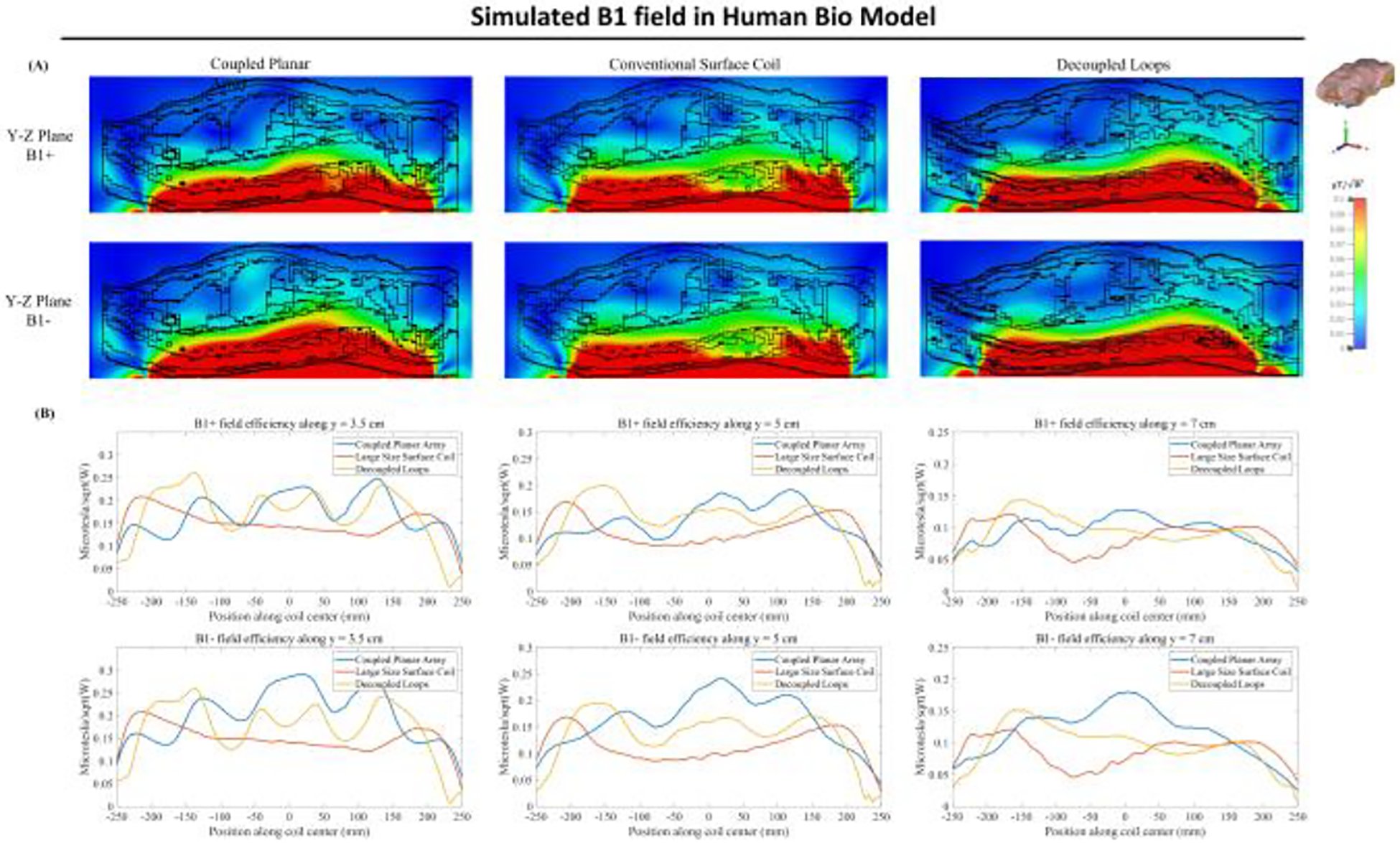
Comparison between simulated B1 field efficiency maps generated by coupled planar array, conventional surface coil, and decoupled loops on the Y-Z plane of the human bio model.

Figure 11 depicts a comparative analysis of the B field efficiency between the simulation model of the coupled planar array, the conventional surface coil, and decoupled loops, each loaded with the CST human bio model named Gustav. Operating at 300 MHz, the B1 field distributions from both coils exhibit resilience against variations in load, maintaining a level of consistency that mirrors the results obtained when an oil phantom is used within the coils. Notably, the B1 field efficiency within the human phantom for the coupled planar array remains higher compared to that of the conventional surface coil and decoupled loops. The coupled planar array effectively sustains high B1 field efficiency, as demonstrated through simulations using an oil phantom for mimicking unloaded conditions and a human bio-model for scenarios representative of potential real imaging applications.

### D. Specific absorption rate

**Fig. 12.**
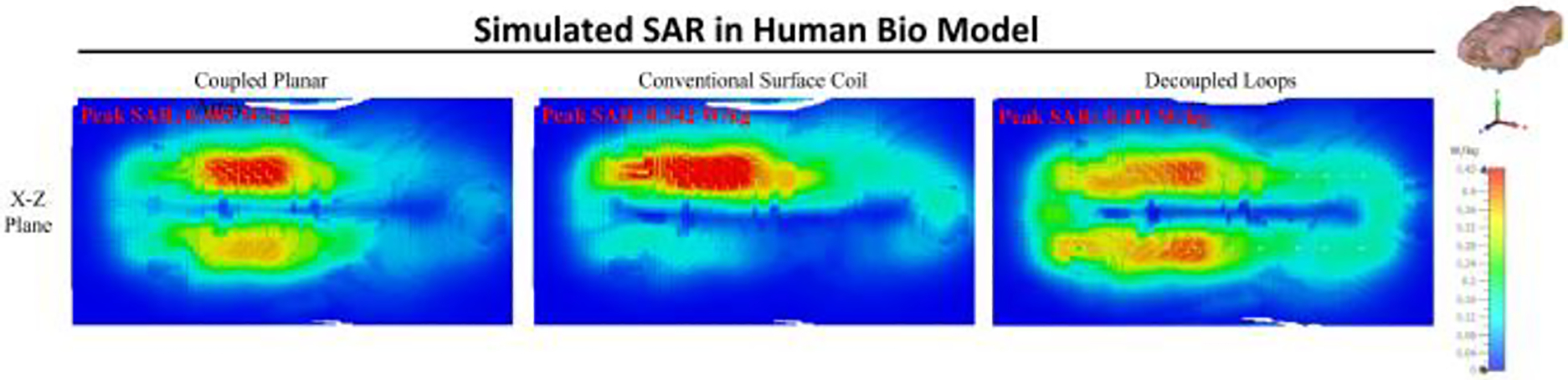
Comparison between Peak SAR generated by coupled planar array, conventional surface coil, and decoupled loops on human bio model

Results of the Specific Absorption Rate (SAR) induced by the coupled planar array and the conventional surface coil are illustrated in Figure 12. This figure demonstrates the simulated SAR values in a human bio model when subjected to both coil types at a frequency of 300 MHz. Simulations with a human bio model at 300 MHz yield peak 10 g average SAR values of 0.485 W/kg for the coupled planar array, 0.542 W/kg for the conventional coil, and approximately 0.451 W/kg for the decoupled loops. The higher SAR observed on the superior side of the body is possibly attributed to the asymmetric distribution of B1 fields in conductive and high permittivity samples at high frequencies, and also attributed to he natural curvature of the bio-model, which positions the upper side closer to the coil, leading to increased energy deposition in that region.

## IV. Discussion

One of the primary challenges in designing large transmit coils at ultrahigh fields is achieving the required high frequency while maintaining resonance stability. Reducing capacitance to increase frequency often results in circuit instability due to increased interaction between the resonant structure and its surrounding environment, as well as augmented electric fields. These interactions make the resonance highly sensitive to the environment factors, leading to frequency shifts and, consequently, operational instability or malfunction. While adding multiple splitting capacitors can mitigate some of these issues, it complicates frequency tuning and significantly reduces the tuning range, especially for large coils. The proposed multimodal coupled planar array technique addresses these challenges by leveraging its highest frequency mode, enabling efficient high-frequency operation without the need for excessively small capacitance values. This design ensures excellent resonance stability, high quality factors, and scalability, offering flexibility and reliability for a wide range of clinical and research applications.

High RF power deposition and patient safety are major concerns in ultrahigh field MRI where the required excitation power is significantly higher compared to low field MRI. This safety concern is even more pronounced in large field-of-view (FOV) imaging, where a large transmit coil is typically used and the required excitation power is further increased in order to excite a large volume of sample. The reduced SAR of the proposed multimodal coupled planar array technique is advantageous and can potentially mitigate the tissue heating problem in ultrahigh field imaging.

Improving the efficiency of the transmit B1 fields (B1+) is another way to reduce the excitation power and thus the power deposition in the imaging sample. Due to their large physical size, conventional L/C loop coils suffer from low B1 efficiency and increased excitation power. The multimodal coupled planar array uses multiple small-sized resonators that are electromagnetically coupled to form a large multimodal resonator. This unique structure with more conductors carrying electric currents helps to enhance the B1 fields in the imaging region. Given the circuit structure described in this paper, it is possible to design a double-tuned large-size transmit coil based on the proposed coupled planar array technique to facilitate heteronuclear metabolic MR imaging and spectroscopy at ultrahigh fields.

A decoupled multichannel overlapped loop array is a common design strategy for achieving large-volume transmission without the need for a parallel transmit (pTx) system. Since the required high power multichannel RF transmitter [41] is not often available in standard or clinical MR scanners, this approach typically relies on a power splitter combined with a Butler matrix to interface to the MR scanner and distribute phase-shifted signals to each element, enabling improved field uniformity. We have performed the comparison, using a decoupled overlapped loop array consisting of five resonant elements fed through a power splitter. As shown in Figure 9, 10, and 11, our study demonstrates that the B1 field efficiency of the proposed coupled multimodal coil is higher than that of the decoupled planar array. It’s worth noting that this result does not include the insertion loss of the required power splitter and Butler matrix in the decoupled multichannel array. A typical power splitter introduces approximately 0.3–0.5 dB loss, and the Butler matrix adds an additional 0.5–1 dB, resulting in a total loss of 17–30% in power, or a reduction of 10–17% in B1 efficiency. These losses negatively impact the overall transmit (and reception if the array is used as a transceiver) efficiency of the system.[42, 43]. In addition, decoupled loop arrays require sufficient electromagnetic decoupling among resonant elements [44–47], particularly between those non-adjacent elements in a transmit or transceiver array. This remains a technical challenge in practical designs at ultrahigh fields [48–50]. In contrast, the proposed coupled multimodal planar array operates as a single-port driven structure and requires no decoupling or complex yet lossy feeding network. This simplicity offers significant practical advantages in designing transmit coils for excitation, especially for scenarios where multichannel transmission system is unavailable or impractical.

**Fig. 13.**
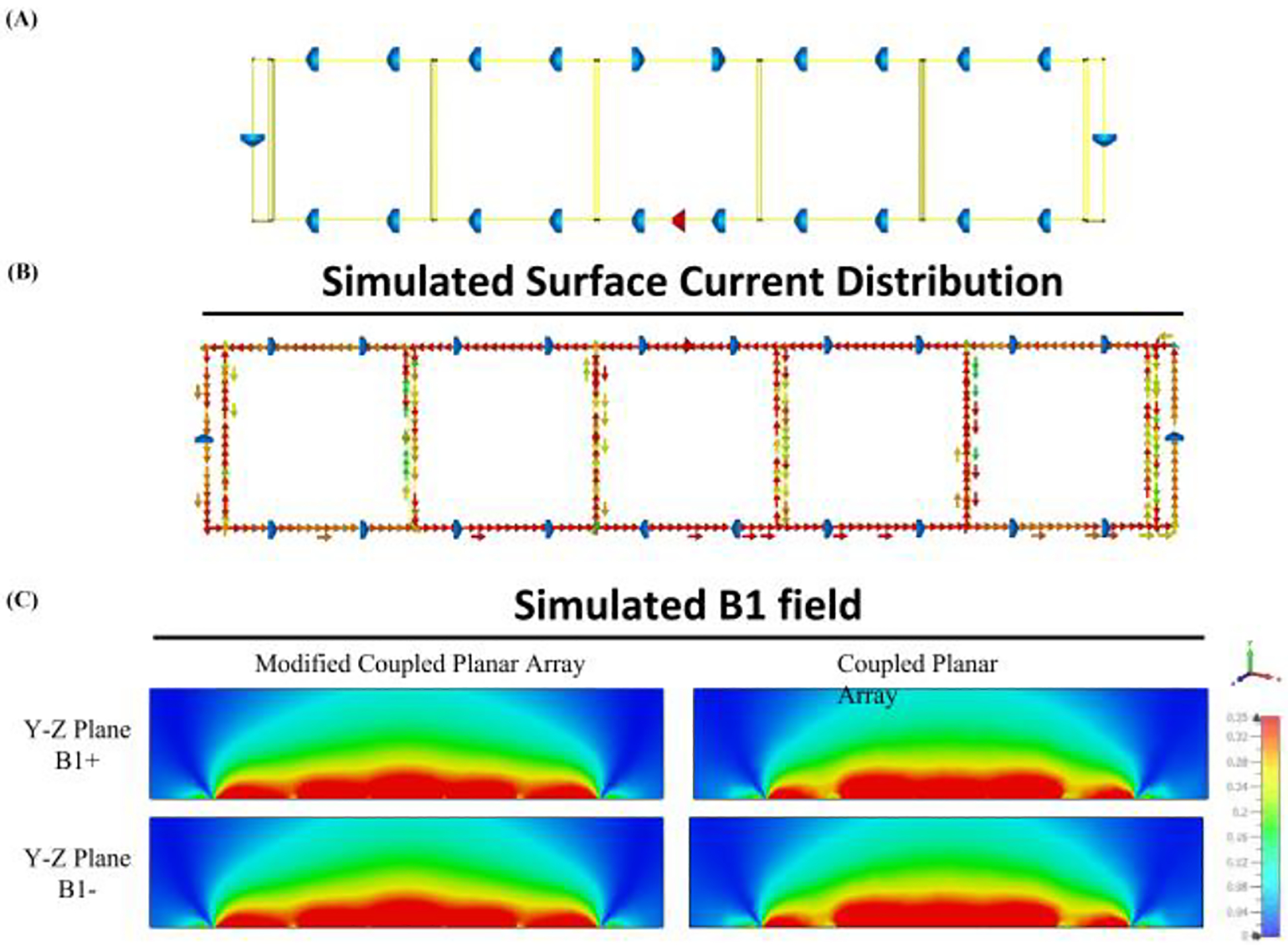
(A) Modified coupled planar array with two 1 cm coils added on the sides. (B) Simulated surface current distribution plot of the modified multimodal surface coil. (C) Simulated B1 field distributions on the YZ-plane.

**Fig. 14.**
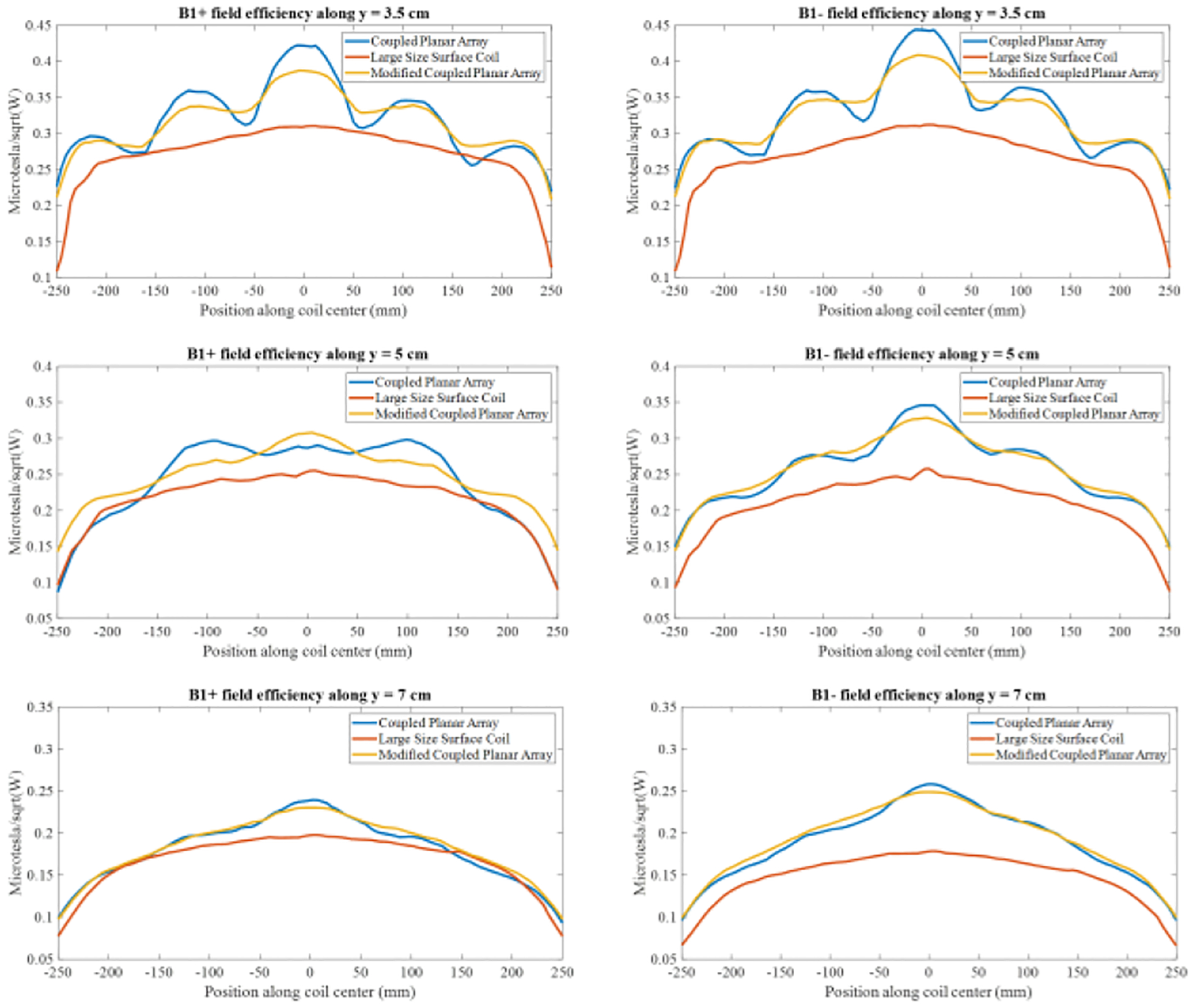
1-D plot of B1 field efficiency along the horizontal line y = 3.5 cm, y = 5cm, and y = 7 cm.

Our results indicate that the B_1_ field at the lateral edges of the original coupled planar array is weaker, reflecting inherently lower current density in the peripheral elements. This reduction in side-lobe strength contributes to diminished in-depth excitation in off-center regions and manifests as transmit inhomogeneity across the imaging volume. To address the observed inhomogeneity in the transmit field, we investigated a modified setup of the coupled planar array by adding two small coils to the sides of the array, as shown in Figure 13A. These additional coils, each 1 cm in width, were tuned to the same frequency as the other coils in the array to ensure consistency in performance. Despite the modification, the coupled planar array remained tuned to 298 MHz at its highest frequency mode. This adjustment was specifically designed to enhance the uniformity of the field distribution. The simulated B1 field distributions on the YZ-plane for the original and modified coupled planar arrays are compared in Figure 13B. The modified setup demonstrates significantly improved uniformity across the region of interest without a substantial decrease in field strength. The inclusion of the two small coils helped redistribute the field, minimizing field variations along the horizontal axis that were prominent in the original design. Additionally, Figure 14 provides 1-D plots of the B1 field efficiency along the horizontal lines at y=3.5 cm, y =5 cm, and y=7 cm, further illustrating the impact of the modifications. While the original coupled planar array shows more pronounced variations, the modified design exhibits a more uniform distribution while maintaining comparable efficiency levels. These results highlight the effectiveness of strategic modifications to the array, such as adjusting the number, spacing, or size of coils, in improving field homogeneity. Future studies will systematically explore these parameters to further optimize the balance between uniformity, efficiency, and scalability.

Both the coupled planar array and classic ladder resonators employ distributed L/C networks to support multiple RF modes, yet they differ markedly in implementation and intended function [51]. Ladder coils, essentially non-closed or partial birdcage structures, possess a large total inductance that limits their achievable resonant frequency, making them unsuitable for large-area, high-frequency operation at ultrahigh fields. In contrast, the proposed coupled multimodal planar array uses tight electromagnetic coupling among smaller loop elements to shift its highest mode into the 300 MHz band without requiring impractically small capacitances. This enables the array to deliver high B_1_ efficiency over a large footprint and achieve efficient, large-area excitation at ultrahigh fields.

## V. Conclusion

In this study, we have successfully developed a high-frequency large-size transmit coil based on the proposed coupled planar array technique for ultrahigh field spine MR imaging. Compared to the conventional loop surface coils with the same size and geometry, the coupled planar array offers superior ability to operate at high frequencies, improved B1 efficiency and penetration, as well as reduced SAR. The coupled planar array technique provides a simple and robust solution to the design of high-frequency large-size transmit coils in spine imaging at ultrahigh fields and ultimately helps enhance the functionality and patient safety of ultrahigh field MRI.

## Figures and Tables

**TABLE I:** Resonant Modes, Frequencies, and Relative Current Patterns of the Coupled Planar Array

Mode	Frequency	Relative Current Pattern
**Mode 1 (Lowest)**	$\omega_{1}=\omega_{0}\frac{1}{\sqrt{1-k\sqrt{3}}}$	+, −, +, −, +
**Mode 2**	$\omega_{2}=\omega_{0}\frac{1}{\sqrt{1-k}}$	+, −, 0, +, −
**Mode 3**	$\omega_{3}=\omega_{0}$	+, 0, −, 0, +
**Mode 4**	$\omega_{4}=\omega_{0}\frac{1}{\sqrt{1+k}}$	+, +, 0, −, −
**Mode 5 (highest-frequency mode; operational mode)**	$\omega_{5}=\omega_{0}\frac{1}{\sqrt{1+k\sqrt{3}}}$	+, +, +, +, +
